# Circulating CD9+/GFAP+/survivin+ exosomes in malignant glioma patients following survivin vaccination

**DOI:** 10.18632/oncotarget.21773

**Published:** 2017-10-10

**Authors:** Phillip M. Galbo, Michael J. Ciesielski, Sheila Figel, Orla Maguire, Jingxin Qiu, Laura Wiltsie, Hans Minderman, Robert A. Fenstermaker

**Affiliations:** ^1^ Department of Neurosurgery, Roswell Park Cancer Institute, Buffalo, New York, 14263 USA; ^2^ Flow and Image Cytometry, Roswell Park Cancer Institute, Buffalo, New York, 14263 USA; ^3^ Pathology, Roswell Park Cancer Institute, Buffalo, New York, 14263 USA; ^4^ Pediatrics, Roswell Park Cancer Institute, Buffalo, New York, 14263 USA; ^5^ Center for Immunotherapy, Roswell Park Cancer Institute, Buffalo, New York, 14263 USA; ^6^ Jacobs School of Medicine and Biomedical Sciences, State University of New York, Buffalo, New York, 14214 USA

**Keywords:** exosome, glial fibrillary acidic protein, glioblastoma, survivin, vaccine

## Abstract

Glioma cells release exosomes in culture and into the extracellular matrix *in vivo*. These nanobodies transport an array of biomolecules and are capable of mediating cell-cell communication. Circulating exosomes in cancer patients may be indicative of disease status and response to therapy. The inhibitor of apoptosis protein (IAP) survivin (SVN) promotes cancer cell proliferation, local immune suppression and resistance to chemotherapy and it is a potential cancer biomarker. We used imaging flow cytometry to perform quantitative measurements of circulating SVN+ exosomes in the serum of malignant glioma patients undergoing investigational treatment with an anti-survivin vaccine (SurVaxM). Serum from glioma patients contained abundant CD9+ exosomes with both SVN and glial fibrillary acidic protein (GFAP) on their surface. Survivin and GFAP were evaluated both independently and together as possible tumor markers on CD9+ exosomes. Patients with longer time to tumor progression generally exhibited a decrease in circulating CD9+/SVN+ and CD9+/GFAP+/SVN+ exosomes immediately following survivin vaccination; whereas, those with early tumor progression had an increase in exosomes, despite anti-survivin immunotherapy. Serum from non-cancer healthy control individuals had very few detectable CD9+/GFAP+/SVN+ exosomes, although CD9+/GFAP+ exosomes were detectable in small numbers. This study demonstrates that patients with malignant gliomas have CD9+/GFAP+/SVN+ and CD9+/SVN+ exosomes that are released into the circulation and that early reductions in their numbers following anti-survivin immunotherapy might be associated with longer progression-free survival.

## INTRODUCTION

Malignant gliomas constitute a group of brain cancers for which advances in treatment have been quite limited. The most common of these cancers, glioblastoma, remains a devastating disease with few long-term survivors. Currently, glioma patients undergoing therapy are followed with serial magnetic resonance imaging studies, but to date no clinically useful liquid biomarkers have been made widely available for monitoring disease status. Several investigators have suggested that analysis of circulating extracellular vesicles or exosomes might provide a means to develop helpful diagnostic tools for evaluating these patients [1–6]. However, the application of existing technology for high throughput assessment of protein biomarkers on the surface of exosomes has been relatively limited.

There is considerable interest in exosomes and other types of extracellular vesicles because of their ability to transport an array of biomolecules between cells, suggesting a potential mechanism for cell-cell communication. Studies indicate that exosomes isolated from glioblastoma cell culture media transport RNA and protein molecules that may promote tumor growth. Glioma-derived exosomes have been shown to stimulate angiogenesis, tumor cell migration [7] and affect glioma cell proliferation [8]. They also appear to modulate tumor invasiveness [9], a common feature of gliomas accounting for their very high local tumor recurrence rates and consequent lethality. Thus, identification of biomolecules displayed on the surface of circulating exosomes could possibly be used to characterize tumor phenotype.

Survivin is a member of the inhibitor of apoptosis protein (IAP) family that is expressed by many tumor types, including malignant gliomas. Expression of survivin in gliomas and other cancers is associated with a poor prognosis [10–13] and refractoriness to chemotherapy [14]. Although it was initially defined as an intracellular molecule, more recently, survivin has been identified on the outer surface of exosomes produced by cervical and prostatic carcinoma cells [15, 16].

Survivin has multiple biological actions and isoforms. It is primarily expressed during the G2/M phase of the cell cycle where it interacts with the mitotic spindle apparatus. As such, it regulates microtubule dynamics during metaphase and anaphase [17, 18]. Hence, cellular depletion of survivin during mitosis can induce aberrant chromosomal segregation, furthering genomic instability and mitotic catastrophe [19]. It also inhibits apoptosis by modulating the function of certain cell death proteases [20]. These properties and others make it a potential target for cancer therapies.

Epitopes of survivin are presented by MHC I complexes on the surface of tumor cells making them immunologically targetable. Patients with cancer, including gliomas, have anti-survivin antibodies [21] and survivin-specific T cells [22] in peripheral blood. Therefore, survivin is immunogenic and its immunogenicity may be enhanced with the aid of survivin mimics [23, 24]. Several studies have looked at active, specific vaccination against survivin to treat various cancers [25–27], including malignant gliomas [28].

Here we show that patients with malignant gliomas have circulating exosomes with both survivin and glial fibrillary acidic protein (GFAP) detectable on their surface using imaging flow cytometry. In the current study, we specifically examined survivin and GFAP on the surface of serum-derived CD9+ exosomes from patients with recurrent malignant glioma who underwent investigational therapy consisting of active specific vaccination against survivin.

## RESULTS

### Patient characteristics

Patient characteristics are listed in Table 1. All evaluable patients in this study had recurrent high grade glioma WHO grade III (1 patient) or IV (7 patients) and had failed standard treatment. Patients had recurrent or progressive disease documented by MRI at the time of entry. Eight of nine patients originally enrolled in the vaccine study received the full complement of 4 prime-boost doses of SurVaxM and 3 patients received at least 1 additional booster dose following the prime-boost phase of treatment. The composition of the survivin vaccine, adverse events and immunologic effects are reported elsewhere [28]. Of the 9 patients entered in this clinical trial, one individual (not included in Table 1) received only 2 doses of vaccine and was excluded from analysis due to early, rapid tumor progression and unavailability for a follow-up blood sample. All patients had measureable disease on brain MRI scans at entry; however, there was a range in the volume of abnormal contrast enhancing tumor tissue on T1-weighted scans at baseline (Table 1).

**Table 1:** Patient characteristics

Patient	Age	Sex	Tumor type^1^	IDH-1 (R132H)	Disease Burden^2^	Survivin+ Cells in Tumor (%)	Doses of Vaccine	PFS (weeks)
1	38	M	G	+	+	22%	4 + 14	208+
2	58	M	G	+	+	1%	4 + 2	88.0
3	57	M	A	+	+	2%	4 + 1	96.4
4	45	F	G	+	+++	10%	4	8.0
5	52	F	G	-	+	7%	4	8.6
6	48	F	G	-	++	8%	4	10.4
7	61	M	G	-	+	15%	4	25.1
8	54	M	G	+	+	4%	4	9.4

^1^G, glioblastoma; A, anaplastic glioma; ^2^Disease burden measured on MRI at first dosing: (−) no measureable contrast enhancement (C.E.); (+), measureable C.E. < 1 cm^3^; (++), >1cm^3^ but ≤ 5 cm^3^ C.E.; (+++), >5 cm^3^ C.E.; PFS: progression-free survival.

### Survivin and GFAP expression by tumor cells

**Figure 1:**
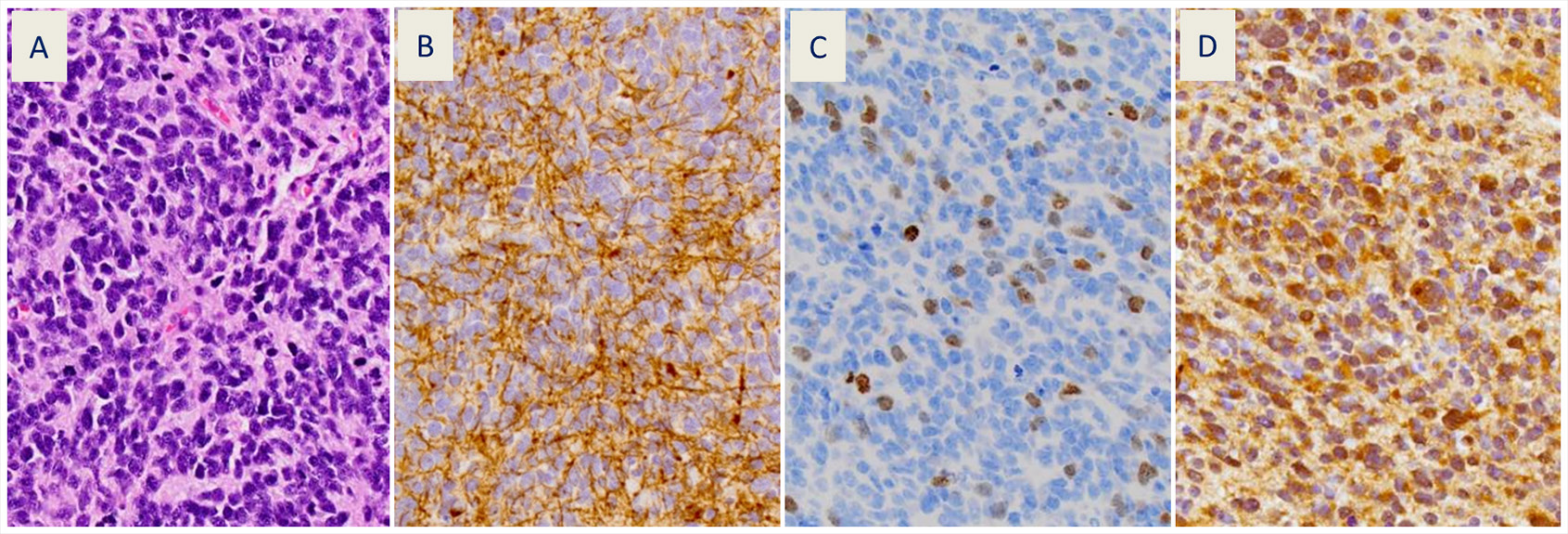
Representative patient tumor sections (patient #1) showing: **(A)** hematoxylin and eosin stain of recurrent glioblastoma with small cell features (400x) and immunohistochemical stains for: **(B)** glial fibrillary acidic protein (GFAP), **(C)** survivin, and **(D)** mutant isocitrate dehydrogenase-1 (IDH-1 R132H).

A diagnosis of either glioblastoma (7 patients) or anaplastic glioma (1 patient) was confirmed (Figure 1A) by the neuropathology co-investigator (J.Q.) in all cases. Of the 8 evaluable patients, all had GFAP (Figure 1B) and survivin (Figure 1C) expression in their tumors, as detected by immunohistochemistry (IHC). GFAP expression was both strong and diffuse in all tumors. Five of eight evaluable patients had IDH-1 mutation R132H (Figure 1D and Table 1). Visually detectable survivin expression was present in 1-22% of tumor cells (Table 1). There was no association between IDH-1 mutation status and survivin expression.

### Exosome characteristics

**Figure 2:**
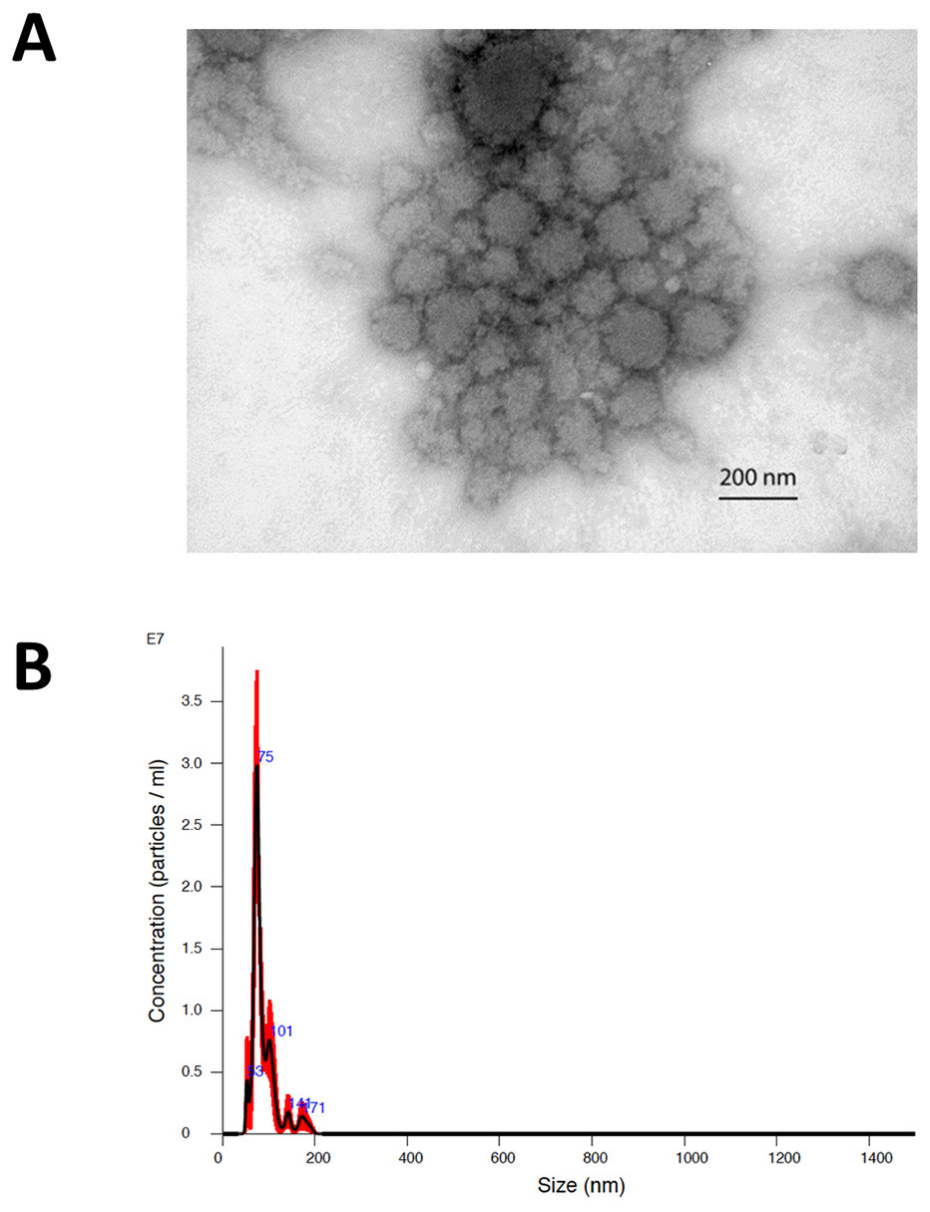
**(A)** Electron microscopic image of exosomes isolated by ultracentrifugation from the baseline serum sample of patient #2. The image was captured at 50,000x and scale bar indicates 200 nm. **(B)** Representative nanoparticle tracking (Nanosight) profile of exosomes isolated by ultracentrifugation (patient #2).

**Figure 3:**
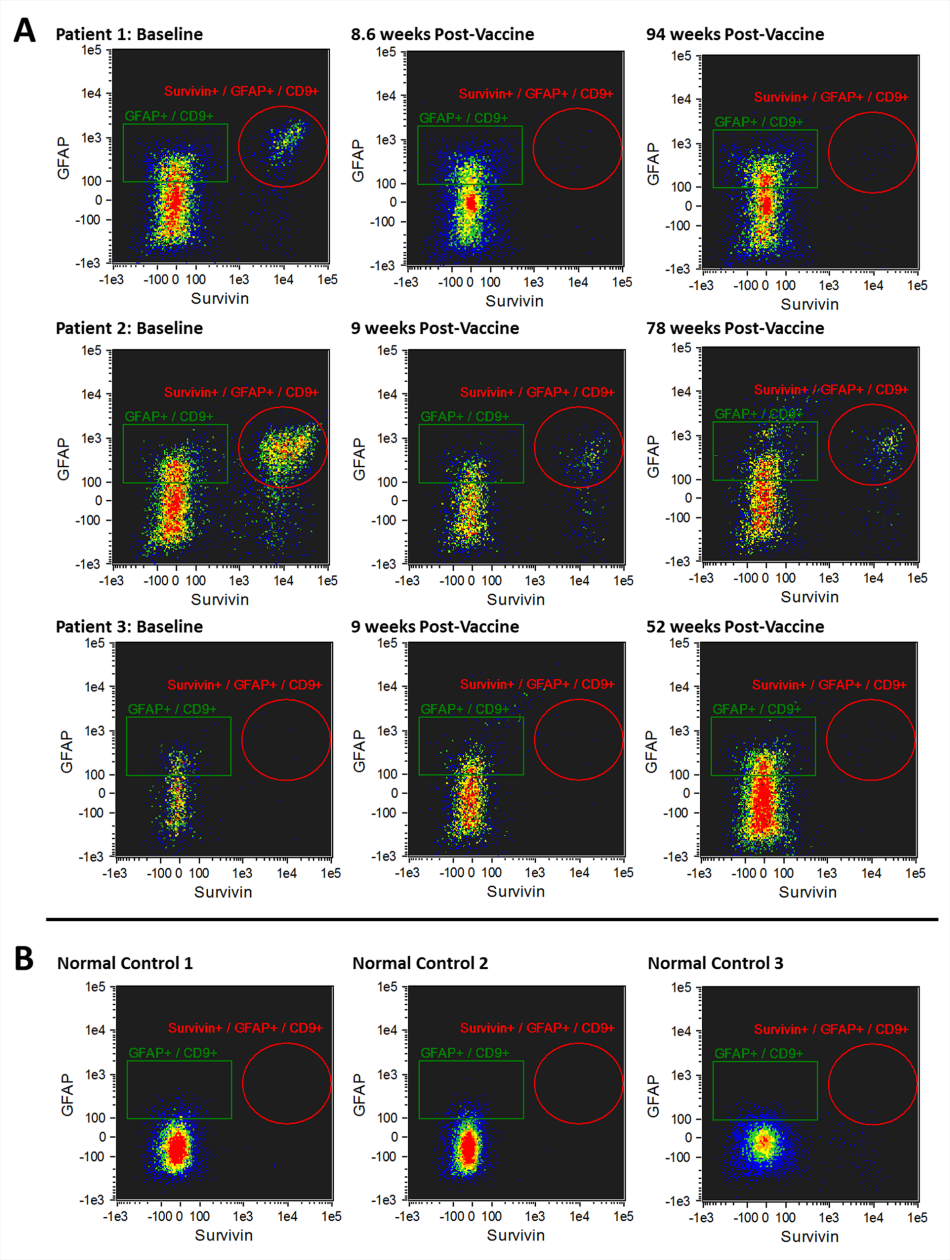
**(A)** Imaging flow cytometry plots of CD9+/GFAP+/SVN+ exosomes in 3 patients who had late tumor progression (88.0-173.3 weeks) after the first of 4 doses of survivin vaccine. CD9+/GFAP+/SVN+ exosomes at baseline (pre-vaccine, left column) are shown; CD9+/GFAP+/SVN+ exosomes 8-10.3 weeks after receiving the first of four vaccine doses (middle column); and CD9+/GFAP+/SVN+ exosomes 12-22 months after receiving first of four doses of survivin vaccine (right column). Bottom row **(B)** demonstrates CD9+/GFAP+/SVN+ exosomes in 3 non-cancer healthy control individuals (no tumor, no vaccine).

**Figure 4:**
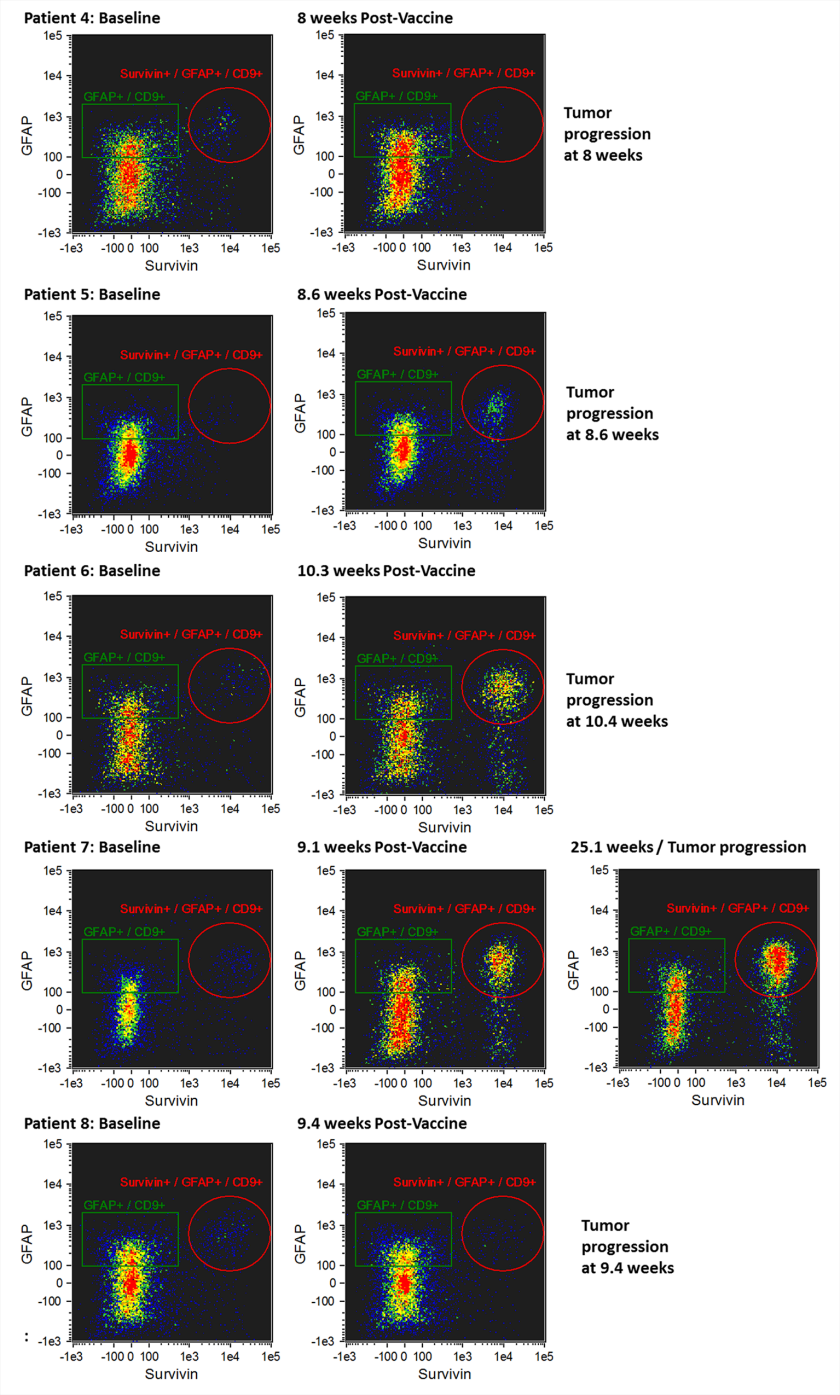
Imaging flow cytometry of CD9+/GFAP+/SVN+ exosomes in patients whose tumors progressed early (8.0-25.1 weeks) following initial vaccination CD9+/GFAP+/SVN+ exosomes at baseline (i.e. prior to first dose of vaccine) are shown in the left column, CD9+/GFAP+/SVN+ exosomes obtained from serum 8-24 weeks after first dose of vaccine (middle column) at the time of tumor progression, and in one patient (#7) at 25 weeks (right panel).

The morphology of exosomes isolated from the serum of patients was characterized using electron microscopy (Figure 2A). In addition, exosome size was measured by nanoparticle tracking analysis (Figure 2B). Results from three captures were averaged, yielding a measured concentration of 3.68 x10^9^ particles/mL, with mean particle size of 87 nm and a modal particle size of 74 nm. GFAP- and survivin-containing, CD9+ exosomes were measured by ImageStream*^X^* imaging flow cytometry in serum samples obtained at baseline (just prior to first vaccination) and approximately 9 weeks (range 8.6 – 10.3 weeks) following the first of four vaccinations administered at 2-week intervals (Figures 3 and 4). In addition to this immediate post-vaccination time point, later follow-up samples were obtained in several patients with relatively longer time to tumor progression (Figure 3). Three non-cancer healthy control individuals who did not receive the survivin immunogen were also assessed for survivin and GFAP expression on CD9+ exosomes (Figure 3, bottom row, and Table 2).

**Table 2:** GFAP+, SVN+ and SVN+/GFAP+ exosomes measured by imaging flow cytometry as a percentage of all CD9+ events

	GFAP+ (% Total CD9+ Exosomes)					Survivin+ (% Total CD9+ Exosomes)					Survivin+/GFAP+ (% Total CD9+ Exosomes)				
**Patient**	**Baseline**	**8 Weeks**	**% change**	**Extended**	**% change**	**Baseline**	**8 Weeks**	**% change**	**Extended**	**% change**	**Baseline**	**8 Weeks**	**% change**	**Extended**	**% change**
1	23.7	25.9	9%	33.1	40%	12.1	0.4	−97%	0.9	−93%	10.4	0.3	−98%	0.6	−94%
2	18.2	16.9	−7%	26.3	45%	35.0	17.1	−51%	11.4	−67%	27.2	11.5	−58%	9.0	−67%
3	22.9	20.5	−10%	18.7	−18%	0.8	1.2	53%	0.3	−63%	0.6	0.4	−31%	0.2	−65%
4	25.0	30.3	21%			5.1	2.0	−61%			3.2	1.2	−63%		
5	19.8	19.4	−2%			1.6	14.1	781%			0.6	9.7	1417%		
6	24.9	19.1	−23%			6.7	31.7	373%			4.4	21.6	393%		
7	21.0	17.8	−15%	16.0	−24%	6.3	27.5	340%	46.5	644%	4.4	19.8	349%	37.6	753%
8	27.1	26.5	−2%			5.3	2.3	−57%			3.8	1.4	−63%		
C1	2.9					0.1					0.0				
C2	3.2					0.0					0.0				
C3	2.7					1.2					0.1				

Glioma patients 1-3 and 4-8 and non-cancer healthy controls (C1, C2 and C3) are shown. Glioma patient #4 is not shown due to failure to complete the prime-boost vaccine regimen due to rapid tumor progression. Baseline measurements were made on serum obtained prior to first vaccination and 8-week values were obtained 2 weeks after administration of the last of 4 vaccine doses.

### Composition of baseline CD9+ exosomes in glioma patients and non-cancer controls

There was no statistically significant difference between the total number of CD9+ exosomes in glioma patients at baseline and the non-cancer control population. In the glioma patients, a relatively large fraction (range 18.2 – 27.1%; mean = 22.8%) of CD9+ exosomes had GFAP on their surface. In contrast, a small but detectable fraction (range 2.7 – 3.2%; mean = 2.9%) of CD9+ exosomes from non-cancer healthy controls had GFAP on their surface (a 7.9-fold difference; Table 2). Taken as a single marker, survivin was present on an average of 9.1% of CD9+ exosomes of glioma patients (range 0.8 – 35%) at baseline (i.e. before vaccination) and on an average of 0.43% of control CD9+ exosomes (range 0.0 – 1.2%; a 21-fold difference; Table 2). The double marker GFAP+/SVN+ subpopulation of CD9+ exosomes was much higher in glioma patients (range 0.6 – 27.2%; mean = 6.8%) than in controls (range 0.0 – 0.1; mean = 0.03%), a 227-fold difference (Table 2). In glioma patients, baseline CD9+/GFAP+/SVN+ exosome levels had no apparent correlation with percentage of survivin-positive cells detected in tumor tissue by immunohistochemistry (Tables 1 and 2). In some cases, however, there was a period of weeks or months between tumor biopsy or resection and study entry (i.e. baseline exosome sampling).

### Late tumor progression

Patients were divided into 2 groups based on time to tumor progression (TTP) > 25.1 weeks arbitrarily defined as late progression (Figure 3) and TTP ≤ 25.1 weeks as early progression (Figure 4). Exosomes were evaluated at baseline (i.e. prior to vaccination) and an average of 9 weeks (8 – 10.3 weeks) following the first survivin vaccination out of a series of 4 vaccinations given over 6 weeks. In patients with late or no tumor progression, two of three individuals experienced tumor progression an average of 15 weeks after initial vaccine treatment (Figure 3 and Table 1). The remaining patient with recurrent glioblastoma remained progression-free beyond 208 weeks (> 4 years).

### Early tumor progression

Five patients experienced early tumor progression with a mean of 12 weeks (8 - 25.1 weeks) from study entry (Figure 4 and Table 2). Early tumor progression was accompanied by an increase in CD9+/GFAP+/SVN+ exosomes (Figure 4). One patient (#7), who was the last of the patients with early progression to progress, experienced a detectable increase in CD9+/GFAP+/SVN+ exosomes 9 weeks following initial vaccination (Figure 4, row 4), which was 16 weeks prior to the detection of tumor progression on brain MRI scanning. This patient was clinically asymptomatic at the time of that scan.

### Antibody 60.11 does not bind the survivin motif contained in the vaccine

**Figure 5:**
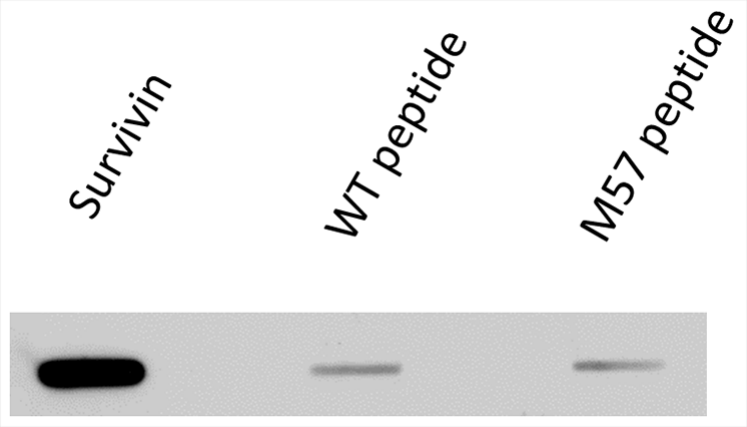
Binding of antibody (60.11) used in imaging flow cytometry to full-length survivin protein and to survivin vaccine peptide aa53-67/M57 and wild type peptide aa53-67

An apparent decrease in survivin-bearing exosomes following vaccination could occur if the detecting antibody recognized the same portion of the survivin molecule that is represented in the survivin peptide vaccine (SurVaxM) itself. Such survivin antibodies produced in response to vaccination could “cloak” the exosomes leading to a failure to detect them by imaging flow cytometry. Antibody 60.11 used in imaging flow cytometry bound to full-length survivin protein immobilized on a filter, but it did not bind to the 15-amino acid long peptide survivin mimic contained in SurVaxM, nor to the corresponding wild type survivin sequence (Figure 5) making this possibility unlikely.

### Relationship of exosome markers to tumor progression

**Figure 6:**
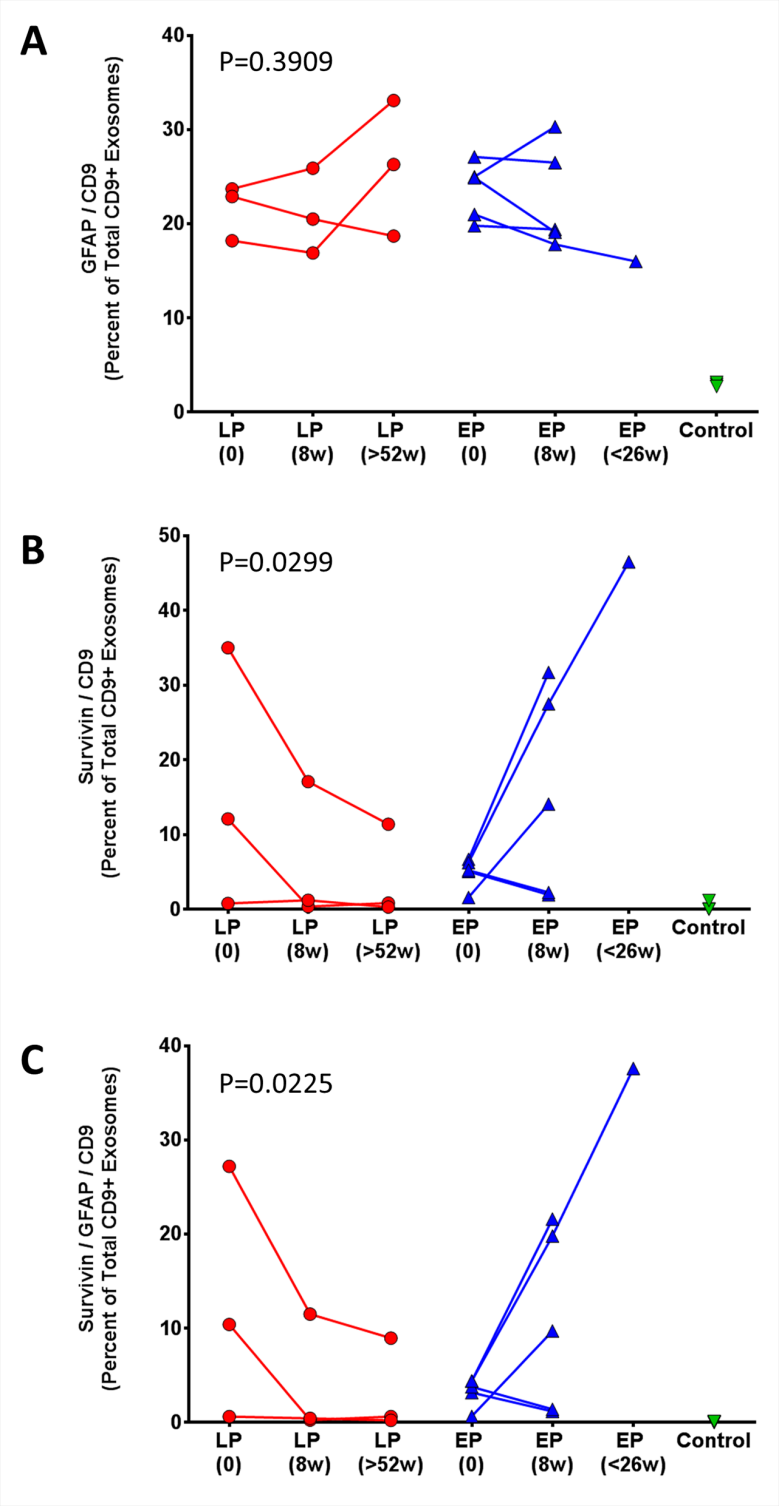
Percent change in **(A)** CD9+/GFAP+, **(B)** CD9+/SVN+, and **(C)** CD9+/GFAP+/SVN+ exosomes following vaccination. Comparison of post-vaccination serum exosomes in patients with: late tumor progression (LP, red) and early tumor progression (EP, blue). Average change in percentage of CD9+/GFAP+ exosomes was assessed with an ordinary two-way ANOVA, p = 0.3909.

Figure 6 show the relationship of the different exosome markers to disease status in patients with early (blue) versus late (red) tumor progression. The fraction of CD9+ exosomes with GFAP marker was variable at 9 weeks (Figure 4A; p = 0.39). In contrast, the fraction of CD9+ exosomes with survivin positivity showed a significant change at 9 weeks, generally consistent with the patient’s tumor status (Figure 6; p = 0.0299). Moreover, the fraction of CD9+ exosomes with both GFAP and survivin positivity also showed a significant change at 9 weeks consistent with the patient’s tumor status (Figure 6; p = 0.0225).

Three patients had either late (20.5 - 22.5 months), or no tumor progression (no evident disease on MRI 4 years after study entry) in one patient (Table 1). This latter patient (#1) without tumor progression experienced a 98% reduction in serum CD9+/GFAP+/SVN+ exosomes 9 weeks after initial vaccination and a 94% reduction at 22 months (Table 2). Late or absent tumor progression was accompanied by a persistent reduction in both CD9+/SVN+ and CD9+/GFAP+/SVN+ exosomes in 2 of 3 patients as well. One patient (#7; Table 2) with late progression experienced an initial increase (+53%) in CD9+/SVN+ exosomes 8 weeks after vaccination; however, both CD9+/SVN+ and CD9+/GFAP+/SVN+ were ultimately lower than baseline at extended follow-up. Moreover, this individual had low survivin expression (2% of tumor cells) by IHC and had exosome counts that were marginally above detectable levels.

## DISCUSSION

Although the blood brain barrier tightly modulates the passage of biomolecules into and out of the central nervous system, it has also been recognized that brain tumor-derived exosomes are found in the bloodstream of patients with malignant gliomas [29]. Exosomes have been shown to cross the intact blood brain barrier [30] and can be used to deliver therapeutic payloads into the brain [30]. Exosomes isolated from the serum of patients with gliomas have been shown to contain EGFR, EGFRvIII, TGF-beta and other proteins and nucleic acid species [1, 2, 31–33]. In one study, various microRNA species, including RNU6-1, were present in serum exosomes from glioblastoma patients, but not non-cancer controls [32]. The possibility that serum exosomes could be used to gauge response to therapy or survival has also been hypothesized [2, 6]. One study has even suggested that microvesicles could help to distinguish between tumor progression and pseudoprogression in glioma patients undergoing treatment [4].

Most studies of exosomes have been conducted using Western blotting and RT-PCR techniques to analyze protein and nucleic acid constituent species, respectively. Because conventional flow cytometers cannot reliably detect extracellular vesicles that are <300 nm, we employed high resolution imaging flow cytometry to obtain a quantitative enumeration of immunolabeled exosomes [34]. This technique is capable of detecting particles in a size range not typically detectable by conventional flow cytometry [35].

CD9 is a member of a family of tetraspanin proteins found in abundance in exosomes. It has been theorized that tetraspanins, including CD9, assist in the biogenesis, cargo selection, and endocytosis of exosomes from the cell [36]. The role of CD9 in cancer development remains a matter of controversy. Among patients with glioblastoma, expression of CD9 by tumor cells has been reported to be associated with a poor prognosis [37]. In our preliminary studies, fluorochrome-conjugated CD9 antibody provided more sensitive detection of isolated exosomes than either CD63 or CD81 antibodies (data not shown). Therefore, CD9 was used as the primary marker for exosome detection here, but other tetraspanins remain to be explored more extensively as well.

Our results indicate that the blood of both glioma patients and non-cancer controls contain a population of CD9+ exosomes. In glioma patients, a relatively large fraction of the CD9+ exosomes were also GFAP-positive (mean = 25.1%); whereas, a small but detectable fraction (mean = 2.9%) of CD9+ exosomes from control individuals had GFAP on their surface (an 8.7-fold difference). Thus, GFAP-containing exosomes can appear in the bloodstream of individuals without glioma making GFAP a relatively non-informative marker by itself. In contrast, survivin was present in 9.1% of CD9+ exosomes of glioma patients and only 0.43% of controls (a 21-fold difference). Moreover, in the CD9+/GFAP+ subpopulation, double-positive exosomes from glioma patients appeared as a much higher percentage of CD9+ exosomes (mean = 6.8%) than in controls (mean = 0.03%), a 227-fold difference. Thus, a combination of markers may be required to provide the best sensitivity and specificity.

GFAP expression is relatively restricted to the central nervous system, although it has also been detected in the renal glomerulus [38] and testis [39] and in osteocytes [40], chondrocytes [41] and fibroblasts [42]. In the CNS, GFAP is expressed by astrocytes where it helps to extend astrocytic processes in support of adjacent neurons [43], maintains blood-brain barrier integrity and supports myelination of white matter [44]. Serum GFAP levels are elevated in different pathologic conditions affecting the CNS, including stroke [45] and tumor [46, 47]. Serum GFAP levels measured by ELISA are reported to be of value in assessing prognosis after closed head injury [48]. While the presence of GFAP in blood following traumatic brain injury (TBI) is thought to occur via the recently identified glymphatic pathway [49], it is not entirely clear whether serum GFAP in these studies is soluble or exosome-associated. Nevertheless, GFAP can be found in the serum of patients with different neuropathologic states and its presence at elevated levels is not specific for the presence of a cerebral glioma. In our study, all non-cancer controls had low but detectable levels of GFAP-containing exosomes in their bloodstream. However, in combination with survivin expression, and viewed within the right clinical context, GFAP may add to the specificity of detection of glioma-derived serum exosomes.

One immunohistochemical study of gliomas revealed that 29 of 29 glioma specimens (WHO grades II-IV) contain survivin-positive cells [12]. In this study, the mean percentage of tumor cells that were survivin-positive cells was 70.0% in low grade gliomas, 81.3% in anaplastic gliomas and 85.0% in glioblastomas. Our patients generally had a lower percentage of cells that stained positively for survivin. In the previous paper, the investigators used polyclonal anti-serum for detection. In contrast, we employed an FDA-approved in-vitro-diagnostic rabbit monoclonal survivin antibody (clone EP119) that appears to be considerably more specific, along with an Omnis (DAKO) autostainer to reduce non-specific staining. The finding of non-uniform survivin expression in tumors is consistent with the fact that survivin expression is cell cycle dependent, appearing largely in G2/M phase [50].

Survivin is found in the nucleus and in mitochondria and has generally been regarded as an intracellular protein with a variety of molecular interactions. More recently, however, the molecule has been identified in association with exosomes produced and released by tumor cells both in culture and *in vivo* [15]. Several actions have been ascribed to exosomal survivin, including adverse effects on immune function within the tumor microenvironment and angiogenic effects as well [51]. Survivin also assists in regulating cell division and inhibiting apoptosis. Although the intracellular functions of survivin are relatively well studied, an understanding of its role in the extracellular matrix and tumor microenvironment is still in a nascent stage.

Survivin is a protein that is strongly tumor-associated. Although it is expressed heavily in fetal tissues [52], expression by adult terminally differentiated tissues appears to be rare. The presence of anti-survivin antibodies [21] and specific T cell responses to survivin-expressing tumor cells in cancer patients strongly suggests that survivin is released systemically from tumor cells and that it is immunogenic to some degree. This is also consistent with evidence that vaccination against survivin appears to stimulate pre-existing immune memory responses in cancer patients leading to the generation of cytotoxic T cell and antibody responses with therapeutic potential [23]. It is possible that survivin on the surface of exosomes is one means by which the molecule is presented to the immune system in cancer patients.

The survivin promoter is known to be inducible by low dose radiation therapy [53]. Moreover, survivin has been identified in exosomes isolated from media of cultured HeLa cells exposed to sub-lethal doses of ionizing radiation [15]. In this study, proton irradiation increased exosomal survivin levels, but did not increase overall exosome numbers. Thus, radiation therapy or chemotherapy, of gliomas could increase survivin levels in serum exosomes, even in the absence of tumor progression. Since radiation therapy is an important component of glioma therapy, it will be important to examine the effects of ionizing radiation therapy and tumor treating fields (TTF) on circulating survivin-containing exosomes.

In the current study, increases in both CD9+/SVN+ and CD9+/GFAP+/SVN+ exosome levels, taken as a fraction of all CD9+ exosomes, proved to be associated with tumor progression; whereas, CD9+/GFAP+ exosomes did not. We did not observe a clear correlation between residual bulk disease on MRI and baseline exosome numbers. Both this, and the weak correlation between exosome counts and tumor cell survivin staining, could be explained by the variable time interval between tumor tissue sampling and baseline exosome recording at study entry.

It is possible that antibodies produced in response to survivin vaccination of glioma patients could have remained bound to serum exosomes resulting in a “cloaking” of survivin moieties on the surface of exosomes measured by imaging flow cytometry. This is not likely to explain the current results since the fluorescent antibody (60.11) used to detect exosomes in imaging flow cytometry does not bind to either the survivin peptide mimic present in the vaccine, or the wild type survivin peptide sequence.

Currently, the best way to monitor patients with malignant gliomas who are undergoing treatment is with brain MRI scans. The combined detection of CD9, survivin and GFAP markers on the surface of serum-derived exosomes from glioma patients by imaging flow cytometry may provide another useful tool for monitoring tumor status in patients receiving survivin-based immunotherapies. It might also be possible to use this technique to monitor glioma progression and response to other types of treatment. The identification of additional tumor markers on the surface of exosomes could enable monitoring of other tumor types, or of distinct populations within them, via sampling of blood. Before utility as a biomarker can be established, it will be essential to assess the independent effects of surgery, radiation-based therapy and chemotherapy on circulating survivin-containing exosomes in larger patient cohorts.

## MATERIALS AND METHODS

### Study overview

The clinical study (NCT01250470), from which blood samples were derived, was conducted in patients with survivin-positive malignant gliomas whose tumors had recurred or progressed following standard therapy. All patients had developed recurrent disease after at least: surgical resection, fractionated radiation therapy and one or more regimens of chemotherapy, including at least temozolomide. Vaccine characteristics and the results of this nonrandomized, single-institution, first-in-man clinical trial designed to assess a fixed-dose, anti-survivin, vaccine regimen have been reported previously [28]. A regimen of SVN53-67/M57-KLH (500 μg) with Montanide ISA 51 and sargramostim (100 μg) was given subcutaneously every two weeks for a total of 4 doses. Patients that survived 6 months without disease progression or regimen-limiting toxicity received additional booster doses of vaccine every three months until tumor progression. Use of the study drug is registered with the FDA under IND #14674. All investigations were performed under a clinical therapeutic protocol approved by the Institutional Review Board at RPCI and in accordance with an assurance filed with the U.S. Department of Health and Human Services. Informed consent was obtained from each subject prior to treatment.

### Patient characteristics and treatment

The study population consisted of patients 18 years of age or older, who had histological proof of recurrent or progressive glioblastoma or anaplastic glioma, following failure of standard therapy. Karnofsky performance status (KPS) ≥ 70, HLA-A*02 or HLA-A*03 haplotype and documented survivin expression by tumor cells were all required for entry. In addition, absence of infection, white blood cell count ≥ 3,000/mm^3^, platelets ≥ 100,000/mm^3^, hemoglobin ≥ 10.0 g/dL, and normal renal and hepatic function were required. Patients were required to use contraceptive methods during and after treatment. Cranial surgery (repeat resection) was permitted prior to entry, but vaccine could not be administered before the 14th post-operative day. Enrolled patients received at least 4 doses of the vaccine to be evaluable for both immunological and clinical response. All patients were followed for immune response and with brain MRI scans to assess tumor response and time to progression.

### Immunohistochemistry

Detection of survivin in surgical tumor specimens was performed with rabbit monoclonal survivin antibody clone EP119 (ready-to-use, Bio SB, Santa Barbara, CA), and IDH-1 (R132H) was detected with mouse anti-human IDH-1 R132H monoclonal antibody clone H09 (1:50, Dianova, Germany) using a Dako Omnis autostainer (Dako North America, Inc. Carpinteria, CA). The percentage of cells expressing survivin was determined by manual counting. IDH-1 mutational status was determined by the presence or absence of cytoplasmic staining.

### Exosome isolation from blood

Patient (n=8) serum samples were collected, processed and stored within 3 hours at −70°C. Blood samples were collected from normal (non-cancer) healthy, non-cancer, control individuals (n=3). Exosomes were isolated from thawed serum by differential ultracentrifugation as previously described (Théry et al, 2006). Serum (200 μl) was spun by ultracentrifuge at 10,000xg for 80 minutes at 4°C in 11 × 34 polycarbonate tubes (Beckman Coulter Inc., Fullerton, California). Supernatant was collected and spun in an ultracentrifuge at 100,000xg for 80 minutes at 4°C using a TLA 100.2 rotor (Beckman Coulter Inc., Fullerton, California). The supernatant was discarded and exosome pellets were re-suspended in 200 μl sterile phosphate buffered saline (PBS). Exosome preparations were stored frozen at −70°C.

### Electron microscopy

Exosomes were isolated and re-suspended in 50 μl PBS, to which 50 μl of 4 % paraformaldehyde was added. Samples were loaded onto Formvar-carbon grids (Electron Microscopy Sciences, Hatfield, PA) as described elsewhere [54]. Grids were incubated on droplets of exosomes for 40 minutes at room temperature, washed once in PBS, and then incubated on droplets of 1 % EM-grade glutaraldehyde for 5 minutes. Grids were stained with UranyLess (EMS, Hatfield, PA), as described in the manufacturer’s instructions. Dried grids were imaged at 80 kV using a JEOL JEM-100CX II Transmission Electron Microscope at the SUNY Buffalo Electron Microscopy Core Facility.

### Antibody specificity

Purified recombinant human survivin (Abcam #ab87202) or survivin peptide (aa 53-67 or aa53-67/M57) were bound to nitrocellulose membrane using the Minifold II slot-blot system (Schleicher & Schuell). Membranes were dried, blocked in 5% milk in TBST for 30 minutes, then incubated overnight with anti-survivin antibody 60.11 (Novus, Littleton, CO) diluted 1:500 in blocking buffer. Membranes were washed in TBST, incubated with HRP-conjugated secondary anti-mouse antibody, washed again, incubated with ECL reagent (Invitrogen), then exposed to X-ray film.

### Exosome labeling and data acquisition

Prior to labeling, conjugated antibodies were spun at 10,000xg to remove antibody aggregates. Exosomes (20 μl) were stained with GFAP Alexa Fluor 488 antibody (BioLegend, San Diego, CA), CD9 PE antibody (BioLegened, San Diego, CA), and survivin DyLight 650 antibody (Novus Biologicals, Littleton, CO) for 30 minutes at 25°C. Data were acquired on an ImageStream^X^ Mark II Imaging Flow Cytometer (AMNIS/Millipore, Billerica, MA). Fluorescent signals were collected as follows: Alexa Fluor 488 was measured at 480-560 nm, Phycoerythrin (PE) was measured at 560-595 nm, and Allophycocyanin (APC) and Dylight 650 were measured at 642-745 nm. All readings were acquired at 60x magnification collected at low flow rate. Data analysis was performed using IDEAS software v6.1. A uniform gating strategy was applied on CD9+ events versus side scatter. Further analysis included GFAP and survivin events based on CD9+ gate. In addition to CD9, serum exosomes were confirmed positive for CD63 and CD81 tetraspanins (data not shown).

### Nanoparticle tracking analysis

Exosome samples were diluted 1×10^6^-fold in PBS immediately prior to nanoparticle tracking analysis. Particle size data were acquired using a NS300 (NanoSight, Malvern Instruments Ltd., Malvern, UK) equipped with a 405nm laser. Thirty-second movies were recorded in triplicate on camera level 15, and then analyzed with detection threshold 5 in NTA 3.2 Build 16.

### Statistical analysis

Exosomes from each patient were compared in terms of the percent increase or decrease over time (approximately 9 weeks following first vaccination) in relation to the raw exosome counts measured in the baseline sample. Analysis of statistical significance was performed using an ordinary two-way ANOVA.

## References

[1] Shi R, Wang PY, Li XY, Chen JX, Li Y, Zhang XZ, Zhang CG, Jiang T, Li WB, Ding W, Cheng SJ (2015). Exosomal levels of miRNA-21 from cerebrospinal fluids associated with poor prognosis and tumor recurrence of glioma patients. Oncotarget.

[2] Muller L, Muller-Haegele S, Mitsuhashi M, Gooding W, Okada H, Whiteside TL (2015). Exosomes isolated from plasma of glioma patients enrolled in a vaccination trial reflect antitumor immune activity and might predict survival. Oncoimmunology.

[3] Evans SM, Putt M, Yang XY, Lustig RA, Martinez-Lage M, Williams D, Desai A, Wolf R, Brem S, Koch CJ (2016). Initial evidence that blood-borne microvesicles are biomarkers for recurrence and survival in newly diagnosed glioblastoma patients. J Neurooncol.

[4] Koch CJ, Lustig RA, Yang XY, Jenkins WT, Wolf RL, Martinez-Lage M, Desai A, Williams D, Evans SM (2014). Microvesicles as a Biomarker for Tumor Progression versus Treatment Effect in Radiation/Temozolomide-Treated Glioblastoma Patients. Transl Oncol.

[5] Santiago-Dieppa DR, Steinberg J, Gonda D, Cheung VJ, Carter BS, Chen CC (2014). Extracellular vesicles as a platform for ’liquid biopsy’ in glioblastoma patients. Expert Rev Mol Diagn.

[6] Gourlay J, Morokoff AP, Luwor RB, Zhu HJ, Kaye AH, Stylli SS (2017). The emergent role of exosomes in glioma. J Clin Neurosci.

[7] Kucharzewska P, Christianson HC, Welch JE, Svensson KJ, Fredlund E, Ringner M, Morgelin M, Bourseau-Guilmain E, Bengzon J, Belting M (2013). Exosomes reflect the hypoxic status of glioma cells and mediate hypoxia-dependent activation of vascular cells during tumor development. Proc Natl Acad Sci U S A.

[8] Skog J, Wurdinger T, van Rijn S, Meijer DH, Gainche L, Sena-Esteves M, Curry WT, Carter BS, Krichevsky AM, Breakefield XO (2008). Glioblastoma microvesicles transport RNA and proteins that promote tumour growth and provide diagnostic biomarkers. Nat Cell Biol.

[9] Hoshino D, Kirkbride KC, Costello K, Clark ES, Sinha S, Grega-Larson N, Tyska MJ, Weaver AM (2013). Exosome secretion is enhanced by invadopodia and drives invasive behavior. Cell Rep.

[10] Chakravarti A, Noll E, Black PM, Finkelstein DF, Finkelstein DM, Dyson NJ, Loeffler JS (2002). Quantitatively determined survivin expression levels are of prognostic value in human gliomas. J Clin Oncol.

[11] Kajiwara Y, Yamasaki F, Hama S, Yahara K, Yoshioka H, Sugiyama K, Arita K, Kurisu K (2003). Expression of survivin in astrocytic tumors: correlation with malignant grade and prognosis. Cancer.

[12] Uematsu M, Ohsawa I, Aokage T, Nishimaki K, Matsumoto K, Takahashi H, Asoh S, Teramoto A, Ohta S (2005). Prognostic significance of the immunohistochemical index of survivin in glioma: a comparative study with the MIB-1 index. J Neurooncol.

[13] Lv S, Dai C, Liu Y, Shi R, Tang Z, Han M, Bian R, Sun B, Wang R (2015). The impact of survivin on prognosis and clinicopathology of glioma patients: a systematic meta-analysis. Mol Neurobiol.

[14] Virrey JJ, Guan S, Li W, Schonthal AH, Chen TC, Hofman FM (2008). Increased survivin expression confers chemoresistance to tumor-associated endothelial cells. Am J Pathol.

[15] Khan S, Jutzy JM, Aspe JR, McGregor DW, Neidigh JW, Wall NR (2011). Survivin is released from cancer cells via exosomes. Apoptosis.

[16] Khan S, Jutzy JM, Valenzuela MM, Turay D, Aspe JR, Ashok A, Mirshahidi S, Mercola D, Lilly MB, Wall NR (2012). Plasma-derived exosomal survivin, a plausible biomarker for early detection of prostate cancer. PLoS One.

[17] Mita AC, Mita MM, Nawrocki ST, Giles FJ (2008). Survivin: key regulator of mitosis and apoptosis and novel target for cancer therapeutics. Clin Cancer Res.

[18] Rosa J, Canovas P, Islam A, Altieri DC, Doxsey SJ (2006). Survivin modulates microtubule dynamics and nucleation throughout the cell cycle. Mol Biol Cell.

[19] Yang D, Welm A, Bishop JM (2004). Cell division and cell survival in the absence of survivin. Proc Natl Acad Sci U S A.

[20] Marusawa H, Matsuzawa S, Welsh K, Zou H, Armstrong R, Tamm I, Reed JC (2003). HBXIP functions as a cofactor of survivin in apoptosis suppression. EMBO J.

[21] Soling A, Plugge EM, Schmitz M, Weigle B, Jacob R, Illert J, Holzhausen HJ, Rainov NG (2007). Autoantibodies to the inhibitor of apoptosis protein survivin in patients with brain tumors. Int J Oncol.

[22] Andersen MH, Pedersen LO, Capeller B, Brocker EB, Becker JC, thor Straten P (2001). Spontaneous cytotoxic T-cell responses against survivin-derived MHC class I-restricted T-cell epitopes in situ as well as ex vivo in cancer patients. Cancer Res.

[23] Ciesielski MJ, Ahluwalia MS, Munich SA, Orton M, Barone T, Chanan-Khan A, Fenstermaker RA (2010). Antitumor cytotoxic T-cell response induced by a survivin peptide mimic. Cancer Immunol Immunother.

[24] NoeDominguez-Romero A, Zamora-Alvarado R, Servin-Blanco R, Perez-Hernandez EG, Castrillon-Rivera LE, Munguia ME, Acero G, Govezensky T, Gevorkian G, Manoutcharian K (2014). Variable epitope library carrying heavily mutated survivin-derived CTL epitope variants as a new class of efficient vaccine immunogen tested in a mouse model of breast cancer. Hum Vaccin Immunother.

[25] Gross S, Lennerz V, Gallerani E, Sessa C, Mach N, Boehm S, Hess D, Boehmer Lv, Knuth A, Ochsenbein A, Gnad-Vogt U, Zieschang J, Forssmann U (2011). First-in-human trial focusing on the immunologic effects of the survivin-derived multiepitope vaccine EMD640744. Journal of Clinical Oncology.

[26] Berinstein NL, Karkada M, Oza AM, Odunsi K, Villella JA, Nemunaitis JJ, Morse MA, Pejovic T, Bentley J, Buyse M, Nigam R, Weir GM, MacDonald LD (2015). Survivin-targeted immunotherapy drives robust polyfunctional T cell generation and differentiation in advanced ovarian cancer patients. Oncoimmunology.

[27] Becker JC, Andersen MH, Hofmeister-Muller V, Wobser M, Frey L, Sandig C, Walter S, Singh-Jasuja H, Kampgen E, Opitz A, Zapatka M, Brocker EB, Thor Straten P (2012). Survivin-specific T-cell reactivity correlates with tumor response and patient survival: a phase-II peptide vaccination trial in metastatic melanoma. Cancer Immunol Immunother.

[28] Fenstermaker RA, Ciesielski MJ, Qiu J, Yang N, Frank CL, Lee KP, Mechtler LR, Belal A, Ahluwalia MS, Hutson AD (2016). Clinical study of a survivin long peptide vaccine (SurVaxM) in patients with recurrent malignant glioma. Cancer Immunol Immunother.

[29] Garcia-Romero N, Carrion-Navarro J, Esteban-Rubio S, Lazaro-Ibanez E, Peris-Celda M, Alonso MM, Guzman-De-Villoria J, Fernandez-Carballal C, de Mendivil AO, Garcia-Duque S, Escobedo-Lucea C, Prat-Acin R, Belda-Iniesta C (2017). DNA sequences within glioma-derived extracellular vesicles can cross the intact blood-brain barrier and be detected in peripheral blood of patients. Oncotarget.

[30] Ha D, Yang N, Nadithe V (2016). Exosomes as therapeutic drug carriers and delivery vehicles across biological membranes: current perspectives and future challenges. Acta Pharm Sin B.

[31] Graner MW, Alzate O, Dechkovskaia AM, Keene JD, Sampson JH, Mitchell DA, Bigner DD (2009). Proteomic and immunologic analyses of brain tumor exosomes. FASEB J.

[32] Manterola L, Guruceaga E, Gallego Perez-Larraya J, Gonzalez-Huarriz M, Jauregui P, Tejada S, Diez-Valle R, Segura V, Sampron N, Barrena C, Ruiz I, Agirre A, Ayuso A (2014). A small noncoding RNA signature found in exosomes of GBM patient serum as a diagnostic tool. Neuro Oncol.

[33] Henriksen M, Johnsen KB, Olesen P, Pilgaard L, Duroux M (2014). MicroRNA expression signatures and their correlation with clinicopathological features in glioblastoma multiforme. Neuromolecular Med.

[34] van der Vlist EJ, Nolte-'t Hoen EN, Stoorvogel W, Arkesteijn GJ, Wauben MH (2012). Fluorescent labeling of nano-sized vesicles released by cells and subsequent quantitative and qualitative analysis by high-resolution flow cytometry. Nat Protoc.

[35] Headland SE, Jones HR, D’Sa AS, Perretti M, Norling LV (2014). Cutting-edge analysis of extracellular microparticles using ImageStream(X) imaging flow cytometry. Sci Rep.

[36] Andreu Z, Yanez-Mo M (2014). Tetraspanins in extracellular vesicle formation and function. Front Immunol.

[37] Podergajs N, Motaln H, Rajcevic U, Verbovsek U, Korsic M, Obad N, Espedal H, Vittori M, Herold-Mende C, Miletic H, Bjerkvig R, Turnsek TL (2016). Transmembrane protein CD9 is glioblastoma biomarker, relevant for maintenance of glioblastoma stem cells. Oncotarget.

[38] Buniatian G, Traub P, Albinus M, Beckers G, Buchmann A, Gebhardt R, Osswald H (1998). The immunoreactivity of glial fibrillary acidic protein in mesangial cells and podocytes of the glomeruli of rat kidney in vivo and in culture. Biol Cell.

[39] Maunoury R, Portier MM, Leonard N, McCormick D (1991). Glial fibrillary acidic protein immunoreactivity in adrenocortical and Leydig cells of the Syrian golden hamster (Mesocricetus auratus). J Neuroimmunol.

[40] Kasantikul V, Shuangshoti S (1989). Positivity to glial fibrillary acidic protein in bone, cartilage, and chordoma. J Surg Oncol.

[41] Kepes JJ, Perentes E (1988). Glial fibrillary acidic protein in chondrocytes of elastic cartilage in the human epiglottis: an immunohistochemical study with polyvalent and monoclonal antibodies. Anat Rec.

[42] Hainfellner JA, Voigtlander T, Strobel T, Mazal PR, Maddalena AS, Aguzzi A, Budka H (2001). Fibroblasts can express glial fibrillary acidic protein (GFAP) in vivo. J Neuropathol Exp Neurol.

[43] Weinstein DE, Shelanski ML, Liem RK (1991). Suppression by antisense mRNA demonstrates a requirement for the glial fibrillary acidic protein in the formation of stable astrocytic processes in response to neurons. J Cell Biol.

[44] Liedtke W, Edelmann W, Bieri PL, Chiu FC, Cowan NJ, Kucherlapati R, Raine CS (1996). GFAP is necessary for the integrity of CNS white matter architecture and long-term maintenance of myelination. Neuron.

[45] Herrmann M, Vos P, Wunderlich MT, de Bruijn CH, Lamers KJ (2000). Release of glial tissue-specific proteins after acute stroke: A comparative analysis of serum concentrations of protein S-100B and glial fibrillary acidic protein. Stroke.

[46] Jung CS, Foerch C, Schanzer A, Heck A, Plate KH, Seifert V, Steinmetz H, Raabe A, Sitzer M (2007). Serum GFAP is a diagnostic marker for glioblastoma multiforme. Brain.

[47] Brommeland T, Rosengren L, Fridlund S, Hennig R, Isaksen V (2007). Serum levels of glial fibrillary acidic protein correlate to tumour volume of high-grade gliomas. Acta Neurol Scand.

[48] Lei J, Gao G, Feng J, Jin Y, Wang C, Mao Q, Jiang J (2015). Glial fibrillary acidic protein as a biomarker in severe traumatic brain injury patients: a prospective cohort study. Crit Care.

[49] Plog BA, Dashnaw ML, Hitomi E, Peng W, Liao Y, Lou N, Deane R, Nedergaard M (2015). Biomarkers of traumatic injury are transported from brain to blood via the glymphatic system. J Neurosci.

[50] Li F, Ambrosini G, Chu EY, Plescia J, Tognin S, Marchisio PC, Altieri DC (1998). Control of apoptosis and mitotic spindle checkpoint by survivin. Nature.

[51] Kucharzewska P, Belting M (2013). Emerging roles of extracellular vesicles in the adaptive response of tumour cells to microenvironmental stress. J Extracell Vesicles.

[52] Adida C, Crotty PL, McGrath J, Berrebi D, Diebold J, Altieri DC (1998). Developmentally regulated expression of the novel cancer anti-apoptosis gene survivin in human and mouse differentiation. Am J Pathol.

[53] Nandi S, Ulasov IV, Tyler MA, Sugihara AQ, Molinero L, Han Y, Zhu ZB, Lesniak MS (2008). Low-dose radiation enhances survivin-mediated virotherapy against malignant glioma stem cells. Cancer Res.

[54] Thery C, Amigorena S, Raposo G, Clayton A (2006). Isolation and characterization of exosomes from cell culture supernatants and biological fluids. Curr Protoc Cell Biol.

